# Predictors of male insemination success in the mosquitofish (*Gambusia holbrooki*)

**DOI:** 10.1002/ece3.1775

**Published:** 2015-10-15

**Authors:** Megan L. Head, Regina Vega‐Trejo, Frances Jacomb, Michael D. Jennions

**Affiliations:** ^1^ Division of Evolution, Ecology and GeneticsResearch School of BiologyAustralian National UniversityActonCanberraAustralian Capital Territory0200Australia

**Keywords:** Correlational selection, insemination success, mate choice, mating success, poeciliid

## Abstract

Identifying targets of selection is key to understanding the evolution of sexually selected behavioral and morphological traits. Many animals have coercive mating, yet little is known about whether and how mate choice operates when these are the dominant mating tactic. Here, we use multivariate selection analysis to examine the direction and shape of selection on male insemination success in the mosquitofish (*Gambusia holbrooki*). We found direct selection on only one of five measured traits, but correlational selection involving all five traits. Larger males with longer gonopodia and with intermediate sperm counts were more likely to inseminate females than smaller males with shorter gonopodia and extreme sperm counts. Our results highlight the need to investigate sexual selection using a multivariate framework even in species that lack complex sexual signals. Further, female choice appears to be important in driving the evolution of male sexual traits in this species where sexual coercion is the dominant mating tactic.

## Introduction

Studies of sexual selection generally focus on species in which males court females and have extravagant ornaments and/or complex courtship displays. Many researchers have adopted a multivariate approach to look at the resultant selection on male traits due to female mate choice (Lande and Arnold 1983; Blows and Brooks 2003). These studies have shown that selection due to female mate preferences is often both nonlinear (e.g., Greene et al. 2000; Brooks et al. 2005; Gerhardt and Brooks 2009) and favors specific trait combinations rather than acting independently on each trait (i.e., there is correlational selection) (e.g., Blows et al. 2003; LeBas et al. 2004; Bentsen et al. 2006; Ower et al. 2013). We therefore now have a good understanding of how sexual selection is mediated by females in such species.

Despite a focus on species with obvious female choice, in many other species sexual coercion is the dominant male mating tactic (reviewed in Cluttonbrock and Parker 1995; Chapman et al. 2003; Arnqvist and Rowe 2005). Males attempt to copulate through physical force and harassment (Cluttonbrock and Parker 1995), and it is expected that females either exert mate choice through mating resistance or mate indiscriminately to reduce the costs of sexual harassment (i.e., convenience polyandry: Thornhill and Alcock 1983) (Eberhard 2002). Well‐known examples include premating struggles in waterstriders (Arnqvist 1992) and the many Poeciliid fishes where males incessantly harass females (Plath et al. 2007). These species rarely exhibit courtship displays or bear ornamental traits. This does not, however, preclude female‐mediated sexual selection on males. For example, female mating resistance has the potential to generate variation in male mating success (Westneat et al. 1990; Wiley and Poston 1996; Jormalainen 1998; Gavrilets and Arnqvist 2001) due to sexual selection on traits that increase males' insemination success (e.g., genital shape in ground beetles, Yakami 2003; and bed bugs, Tadler 1999), and females might still actively bias male mating success by preferentially associating with particular males (e.g., Japanese macaque, Soltis et al. 1997).

Relatively little is known about the targets or form of sexual selection on males in species with coercive mating systems, or the extent to which female mating preferences influence male reproductive success (Kokko 2005; Muller et al. 2011). Here, we investigate sexual selection on male eastern mosquitofish (*Gambusia holbrooki*), a species of poecillid fish in which males mate solely using a coercive tactic called “gonopodial thrusting”. The gonopodium is a modified anal fin that acts as an intromittent organ. Males stealthily approach females from behind and then dart forward and attempt to insert the tip of the gonopodium into the female's genital opening (Langerhans 2011). We use standard multivariate selection analysis (Lande and Arnold 1983; Blows and Brooks 2003) to determine which male traits are correlated with insemination success, and the apparent direction and shape of selection on these traits. We focus our analysis on morphological and behavioral traits that have been shown to play important roles at various stages of mating. Further, we specifically explore insemination success in the absence of direct male–male competition to isolate effects of male–female interactions on male reproductive success.

Poecillid fish are known for their substantial variation in male body size and have become a model system for understanding how sexual selection drives such variation. It was originally assumed that female mate choice had little role in determining male mating success in *G. holbrooki* (Farr 1989), but later studies suggested that the probability of insemination is influenced by the amount of time since females have mated (Pilastro et al. 1997) and that females can influence the likelihood that forced copulation attempts result in actual genital contact under different environmental conditions (Condon and Wilson 2006). These findings suggest that females exert partial control over whether or not they mate. In addition, previous studies of *G. holbrooki* have shown that females prefer to associate with larger males (e.g., McPeek 1992; Bisazza et al. 2001; Kahn et al. 2012; but see: Bisazza and Marin 1991, 1995). Greater association time might increase mating success for large males if it increases access to females (Bisazza and Marin 1991; McPeek 1992). Larger males can also dominate their rivals for access to females (Bisazza and Marin 1991). However, in the absence of competitors, smaller males attempt more copulations than do larger males (Pilastro et al. 2003), and males that are relatively smaller than females have greater insemination success (Pilastro et al. 1997). Even so, it is still unclear whether this relationship is driven by female size, male size, or both (see fig. 1b of Pilastro et al. 1997). The seemingly higher mating success of small males has been suggested to result from their greater stealth and maneuverability (Bisazza and Marin 1995; Pilastro et al. 1997) but female size varies widely in *G. holbrooki* so the advantage of small male size could reflect the ability to sneak up on females who are *relatively* larger rather than effects due to *absolute* male size. The effects of gonopodium length on male mating success have received less attention, but studies of two *Gambusia* species indicate that females prefer longer gonopodia (*G. holbrooki*: Kahn et al. 2010; *G. affinis*: Langerhans et al. 2005). However, gonopodium length might also reduce male maneuverability: Male *G. affinis* with longer gonopodia relative to their body size have slower burst swimming speed (Langerhans et al. 2005). Here, we extend previous work by taking a multivariate approach to examining how male traits influence insemination success.

We predict that if male insemination success is primarily driven by male adeptness at coercion then smaller males will be more likely to inseminate females. Unlike previous studies that report a correlation between male size and insemination success (Pilastro et al. 1997), here we experimentally control female body size to isolate the effect of absolute male size. Alternatively, if insemination success is driven by female preferences, we predict that large males, and those with longer gonopodia will be more successful. If both processes operate, however, they might cancel each other out so that neither body nor gonopodium size have a detectable effect on insemination success.

## Methods

### Origin and maintenance of fish

Test females were offspring of wild‐caught females collected in Canberra, Australia, in March 2013. Females were housed in single sex tanks (30–60 fish per 90 L) to ensure virginity. Males were collected from the wild in February 2014 and kept in the laboratory for 3–6 months prior to our experiment. All fish were maintained at 27°C on a 14:10 light:dark cycle and fed *Artemia salina* nauplii and commercial fish flakes twice daily. Test males were selected haphazardly from stock tanks to reflect the natural size distribution (standard length, SL range: 20.47–26.99 mm).

### Experimental protocol

Prior to each experimental trial, males were stripped of sperm (details below) to fully deplete their sperm reserves then placed in a 7‐L aquarium (17 × 28 × 15 cm) that was divided in two by a mesh barrier. On the other side of the barrier, we placed a stimulus female to prime sperm production (see Bozynski and Liley 2003). Stimulus females varied in size, but our previous work showed that female size does not influence sperm priming in *G. holbrooki* (Head et al. 2015).

After 3 days, we stripped the male again to estimate his sperm count (3 days is enough time for males on our laboratory diet to replenish their sperm reserves (O'Dea et al. 2014)). After a further 3 days, we then replaced the stimulus female with a standard‐sized female (350–450 g) and recorded the male's mating behavior. The sperm number on day 3 (i.e., after the first 3 days) is our best estimate of the likely amount of sperm a male had available for insemination at the start of his mating trial (i.e., based on replenishment 3 days after being stripped). Test females in mating trials were kept individually in 1‐L tanks for 6 days prior to being introduced to a test male.

To begin a behavioral trial, we removed the mesh barrier and allowed the pair to interact. After two minutes, we began to record their behavior for 10 min. We recorded the time that a male spent following the female and how many mating attempts he directed toward her (see: Vega‐Trejo et al. 2014). After another 20 min, both fish were removed from the tank, and we attempted to collect sperm from the test female's reproductive tract. In total, we ran 58 mating trials.

### Collecting and counting sperm from males

Sperm were stripped from males following the methods of Matthews et al. (1997). Briefly, following anesthesia in an ice slurry, males were placed on their side on a glass slide under a dissecting microscope. The gonopodium was swung forward and pressure was gently applied to the abdomen to expel sperm. Using a 10‐*μ*L pipette, we transferred the stripped ejaculate to a microcentrifuge tube containing a known volume (100–300 *μ*L) of saline solution (0.9% NaCl).

We counted sperm following the methods in Evans (Evans 2009). Briefly, samples were vortexed for one minute to break up sperm bundles and evenly distribute sperm throughout the sample. Then, 10 *μ*L of the sample was placed on a Neubauer hemocytometer under ×400 magnification (Kiyowa, Medilux‐12 microscope). We photographed five cells of the hemocytometer so that sperm could later be counted blind to treatment. The five counts were summed, and the total number of sperm per fish was calculated.

### Collecting sperm from females

Within 10 min of the pair being separated, we anesthetized the female in an ice slurry and retrieved sperm, if present, from her gonoduct (see: Pilastro et al. 1997; Pilastro and Bisazza 1999). The female was placed ventral side up on a cradle under a dissecting microscope. A glass micropipette was then used to flush her gonoduct with 30 *μ*L of saline solution (0.9% NaCl). We then vortexed the sample for 60 sec to break up sperm bundles and evenly distribute sperm throughout the sample. We placed 10 *μ*L on a Neubauer hemocytometer for viewing under ×400 magnification (Kiyowa, Medilux‐12 microscope). The presence or absence of sperm was recorded.

### Data analysis

We used a standard multivariate selection analysis to estimate linear and nonlinear sexual selection on male phenotypes (Lande and Arnold 1983). We assigned males an absolute fitness score of 1 or 0 depending on whether or not sperm was extracted from his test female. This absolute fitness score was transformed to relative fitness by dividing by the mean fitness calculated across the experiment (Lande and Arnold 1983). We then fitted a linear regression model including five male phenotypic traits (body length, gonopodium length, estimated sperm number, time spent following the female, and number of mating attempts) as predictor variables and relative fitness as the response variable to estimate the vector of standardized linear selection gradients (*β*). All male traits were standardized (mean = 0; standard deviation = 1). A quadratic regression model including all the linear, quadratic, and cross‐product terms was fitted to estimate the matrix of standardized nonlinear selection gradients (*γ*). To reflect actual selection, the quadratic regression coefficients were doubled (see: Stinchcombe et al. 2008).

Relative fitness was binomially distributed. This does not influence the sign or magnitude of selection gradients (Lande and Arnold 1983), but it presents problems with testing the significance of these gradients. Therefore, to assess the significance of our linear and nonlinear selection gradients, we used GLM with a quasibinomial error structure (Fairbairn and Preziosi 1996; Gershman et al. 2014). To test the overall contribution of linear and nonlinear effects in our models, we used partial *F*‐tests (Chenoweth and Blows 2005). We also calculated phenotypic correlations between male traits. All statistical tests were run in R version 3.2.0 (R development core team 2012).

## Results

Phenotypic correlations between male traits indicate that larger males had longer gonopodia, and that males that spent more time following females made more mating attempts (Table 1). There was also a nonsignificant trend for males with a longer gonopodia to have more sperm.

**Table 1:** Below the diagonal, the vector of standardized linear selection gradients (*β*) and the matrix of standardized quadratic and correlational selection gradients (*γ*) for male phenotypic traits in *Gambusia holbrooki* (Significance was determined using GLM with a quasibinomial error structure). Above the diagonal (shaded) are the phenotypic correlations between traits. Estimates are followed by *P*‐values in brackets

	*β*	*γ*				
		Body length	Gonopodium length	Sperm number	Time following	Mating attempts
Body length	0.165 (0.324)	1.026 (0.073)	**0.664 (0.000)**	0.124 (0.356)	0.125 (0.349)	0.186 (0.162)
Gonopodium length	−0.043 (0.850)	−0.810 **(0.015)**	**1.398 (0.025)**	0.251 (0.057)	0.005 (0.970)	0.083 (0.534)
Sperm number	0.050 (0.797)	0.205 **(0.037)**	0.324 (0.928)	−**1.25 (0.022)**	−0.019 (0.886)	0.130 (0.332)
Time following	−**0.424 (0.038)**	−0.207 (0.577)	0.157 (0.287)	−**0.583 (0.044)**	0.240 (0.458)	**0.709 (0.000)**
Mating attempts	0.272 (0.121)	0.041 (0.810)	0.153 (0.527)	**0.422 (0.038)**	−0.065 (0.359)	0.094 (0.359)

Bold values are statistically significant.

**Figure 1:**
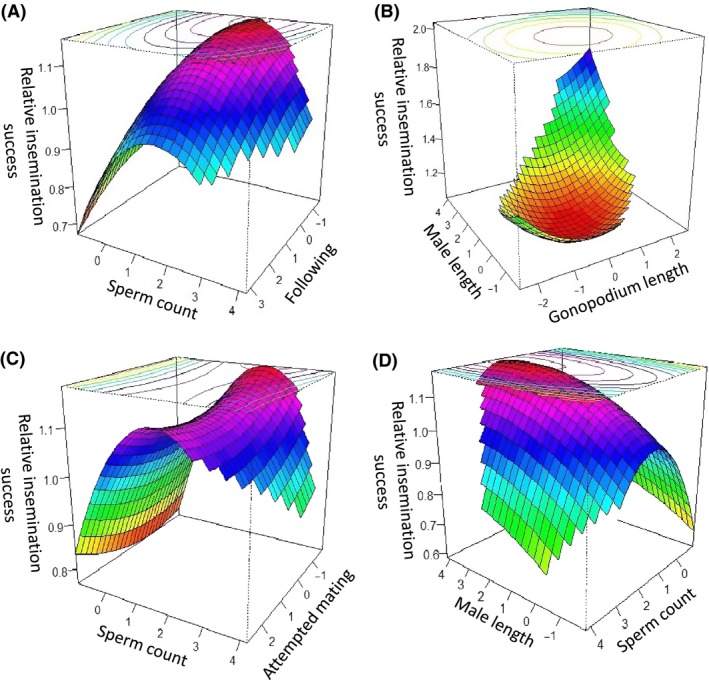
Response surfaces showing correlational selection. (A) the predicted relationship between sperm count, following and relative insemination success, (B) the predicted relationship between male length, gonopodium length, and relative insemination success, (C) the predicted relationship between sperm count, number of male mating attempts, and relative insemination success, (D) the predicted relationship between sperm count, male length, and relative insemination success. All phenotypic traits are standardized.

In total, we retrieved sperm from 31 of the 58 test females. Although our relatively low sample size means that we have low statistical power our selection analysis using insemination success as the measure of fitness showed that, overall, linear selection did not significantly improve the fit of our model (partial *F* test: *F*_(5,52)_ = 1.289, *P* = 0.283), but that nonlinear selection did (partial *F* test: *F*_(15,37)_ = 2.0297, *P* = 0.040). Looking at individual male traits in our analysis showed that males that followed females for longer were significantly less likely to be successful (Table 1, Fig. 1A). There was also significant disruptive selection on male gonopodia length, and significant correlational selection due to an interaction between male body size and gonopodia length (Table 1). Large males with long gonopodia were more successful at inseminating females than were small males with short gonopodia (Fig. 1B).

There was stabilizing selection on the number of sperm, as well as correlational selection on sperm number due to interactions with the time spent following a female, the number of mating attempts, and body length. These selection gradients can be visualized in Figure 1. They show that males that produce intermediate amounts of sperm were relatively more successful at inseminating females if: (1) they had low rates of following (Fig. 1A); (2) made few mating attempts (Fig. 1C); (3) were large males (Fig. 1D).

## Discussion

Here, we examine selection on male traits predicted to affect male insemination success in *Gambusia holbrooki,* a species whose mating system is dominated by sexual coercion. We found no evidence for linear selection on four of the five traits we measured; however, there was both quadratic selection and correlational selection involving all five traits. Notably, large males with long gonopodia were significantly more likely to inseminate females than their counterparts. These results, combined with those of Kahn et al. (2010) showing that females prefer larger males and males with longer gonopodia, are consistent with females mediating male insemination success in *G. holbrooki*. Like studies of selection on complex sexual displays (e.g., Blows et al. 2003), our findings highlight the importance of examining multivariate selection on sexual traits.

### Body size and gonopodium length

Male body size is a trait that often affects multiple mechanisms of sexual selection, a factor which might contribute to generally higher variation in male than female body size in many species (Wyman and Rowe 2014). In poeciliids, three mechanisms of sexual selection operate on male body size: male–male competition, sexual coercion, and female choice. Our finding that large males with long gonopodia were more likely than small males with short gonopodia to inseminate females suggests that insemination success in *G. holbrooki* is driven more by female mate preferences than a male's ability to force copulations. We did not directly tease apart the effects of sexual coercion and female mate choice on insemination success because these two mechanisms of selection occur simultaneously. However, the net selection for large males with large gonopodiums suggests that selection resulting from female choice overrides selection resulting from sexual coercion. We make this claim because Kahn et al. (2010) showed that females prefer to associate with large males that have longer gonopodia. This matches our own findings of which males were most successful. It is suggestive that female choice is still an important selective pressure in mating systems seemingly dominated by male sexual coercion (Eberhard 2002). Other studies that have teased apart the effects of sexual coercion and mate choice (e.g., Sih et al. 2002; Hall et al. 2008) also showed that female choice and sexual coercion act in opposing directions and that when mate choice occurs net selection on male traits is altered.

Previous studies have shown that large males dominate access to females when males compete directly (e.g., Bisazza and Marin 1995; Booksmythe et al. 2013). In our study, we deliberately excluded male–male interactions and found that larger males were more likely to inseminate females. Thus, sexual selection due to direct male–male competition and insemination success in the absence of rivals appear to act in concert, favoring larger males. This finding appears to be consistent across a variety of taxa (reviewed in Hunt et al. 2009). In contrast, however, Pilastro et al. (1997) found that a larger absolute difference in male and female size (i.e., relatively smaller males) increased the likelihood that a female was inseminated, suggesting that male–male competition and insemination ability create opposing sexual selection on male size. This difference between our study and that of Pilastro et al. (1997) could result from environmental differences that potentially influence the relative importance of selection arising due to sexual coercion and female choice (e.g., Sih et al. 2002). Testing for such environmental effects will be an interesting avenue for future research.

### Behavior

Male following behavior was negatively related to insemination success. Although counterintuitive this relationship could arise if males pursue females less if they gain a successful insemination (e.g., a refractory period of decreased male sexual activity after mating has been shown in guppies (Pilastro and Bisazza 1999)). Our result indicates the potential for convenience polyandry (sensu Thornhill and Alcock 1983) and male sexual harassment to coevolve in *G. holbrooki*. Females could mate with males to reduce the level of harassment experienced, which could, in turn, select for increased harassment. The advantages of convenience polyandry depend, however, on the costs of mating such as an increased risk of contracting sexually transmitted diseases (STDs) (Lockhart et al. 1996), potential for injury (Crudgington and Siva‐Jothy 2000; Blanckenhorn et al. 2002), or males transferring harmful substances in their ejaculates (e.g., Wigby and Chapman 2005). While there has been a lot of work on the costs of harassment for female poeciliids (including *Gambusia* spp (Plath et al. 2003)), the costs of mating are still poorly studied and deserve more attention. For instance, it would be interesting to test whether gonopodial intromission damages the female reproductive tract (Constantz 1984), or to test for sexually transmitted diseases, which are common in internally fertilizers (Lockhart et al. 1996).

### Sperm count

We found nonlinear selection on male sperm number. Males that produced an intermediate amount of sperm after 3 days were significantly more likely to inseminate females than those producing higher or lower amounts. This was unexpected. Why should males with large amounts of sperm have lower insemination success? One potential explanation is that sperm number is related to another unmeasured trait that also affects insemination success. This caveat about ‘missing traits’ is a limitation common to all selection analyses, as they are correlational and observed relationships between a trait and fitness are not necessarily causal (Ower et al. 2013). For example, a paternity study in guppies found negative directional selection on sperm production, which possibly represented a trade‐off between pre‐ and postcopulatory traits under sexual selection (Head et al. 2008). Clearly, more work is needed to understand why, and how often, sperm number is not positively related to reproductive success in poecillid fishes.

## Conclusions

Many of the male traits we measured were under correlational, but not directional, selection. This is unsurprising given that insemination success is determined by multiple mechanisms of sexual selection. The presence of correlational selection matters. It can have major consequences for the evolution of sexual traits and reproductive tactics. Correlational selection can drive the evolution of suites of integrated traits (Han and Brooks 2013), and build linkage disequilibrium between traits that are influenced by different genetic loci (Price and Langen 1992; Falconer and Mackay 1996; Lynch and Walsh 1998). Consequently, when selection drives a change in one trait, genetically correlated traits co‐evolve (McGlothlin et al. 2005). Correlational selection could promote the evolution of alternative male reproductive tactics that are associated with certain male phenotypes leading, for example, to the evolution of small coercive males and large attractive males (seen in many species). Here, using an underutilized approach to remove direct male–male competition, we show that female choice could play an important role in driving the evolution of such reproductive tactics, even in species where ostensibly the only route to mating success is through male coercion.

## Conflict of Interest

None declared.

## References

[ece31775-bib-0001] Arnqvist, G. 1992 Pre‐copulatory fighting in a water‐strider: inter‐sexual conflict or mate assessment? Anim. Behav. 43:559–567.

[ece31775-bib-0002] Arnqvist, G. , and L. Rowe . 2005 Sexual conflict. Princeton Univ. Press, Princeton, NJ.

[ece31775-bib-0003] Bentsen, C. L. , J. Hunt , M. D. Jennions , and R. Brooks . 2006 Complex multivariate sexual selection on male acoustic signaling in a wild population of *Teleogryllus commodus* . Am. Nat. 167:E102–E116. 16670989 10.1086/501376

[ece31775-bib-0004] Bisazza, A. , and G. Marin . 1991 Male size and female mate choice in the Eastern mosquitofish (*Gambusia holbrooki*, Poeciliidae). Copeia 1991:730–735.

[ece31775-bib-0005] Bisazza, A. , and G. Marin . 1995 Sexual selection and sexual size dimorphism in the Eastern mosquitofish *Gambusia holbrooki* (Pisces Poeciliidae). Ethol. Ecol. Evol. 7:169–183.

[ece31775-bib-0006] Bisazza, A. , G. Vaccari , and A. Pilastro . 2001 Female mate choice in a mating system dominated by male sexual coercion. Behav. Ecol. 12:59–64.

[ece31775-bib-0008] Blanckenhorn, W. U. , D. J. Hosken , O. Y. Martin , C. Reim , Y. Teuschl , and P. I. Ward . 2002 The costs of copulating in the dung fly *Sepsis cynipsea* . Behav. Ecol. 13:353–358.

[ece31775-bib-0009] Blows, M. W. , and R. Brooks . 2003 Measuring nonlinear selection. Am. Nat. 162:815–820. 14737718 10.1086/378905

[ece31775-bib-0010] Blows, M. W. , R. Brooks , and P. G. Kraft . 2003 Exploring complex fitness surfaces: multiple ornamentation and polymorphism in male guppies. Evolution 57:1622–1630. 12940366 10.1111/j.0014-3820.2003.tb00369.x

[ece31775-bib-0011] Booksmythe, I. , P. R. Y. Backwell , and M. D. Jennions . 2013 Competitor size, male mating success and mate choice in Eastern mosquitofish, *Gambusia holbrooki* . Anim. Behav. 85:371–375.

[ece31775-bib-0012] Bozynski, C. C. , and N. R. Liley . 2003 The effect of female presence on spermiation, and of male sexual activity on ‘ready’ sperm in the male guppy. Anim. Behav. 65:53–58.

[ece31775-bib-0013] Brooks, R. , J. Hunt , M. W. Blows , M. J. Smith , L. F. Bussiere , and M. D. Jennions . 2005 Experimental evidence for multivariate stabilizing sexual selection. Evolution 59:871–880. 15926696

[ece31775-bib-0014] Chapman, T. , G. Arnqvist , J. Bangham , and L. Rowe . 2003 Sexual conflict. Trends Ecol. Evol. 18:41–47.

[ece31775-bib-0015] Chenoweth, S. F. , and M. W. Blows . 2005 Contrasting mutual sexual selection on homologous signal traits in *Drosophila serrata* . Am. Nat. 165:281–289. 15729657 10.1086/427271

[ece31775-bib-0016] Cluttonbrock, T. H. , and G. A. Parker . 1995 Sexual coercion in animal societies. Anim. Behav. 49:1345–1365.

[ece31775-bib-0017] Condon, C. H. L. , and R. S. Wilson . 2006 Effect of thermal acclimation on female resistance to forced matings in the Eastern mosquitofish. Anim. Behav. 72:585–593.

[ece31775-bib-0018] Constantz, G. D. 1984 Sperm competition in Poeciliid fishes Pp. 465–485 *in* Smith R. L., ed. Sperm competition and the evolution of animal mating system. Academic Press, New York, NY.

[ece31775-bib-0019] Crudgington, H. S. , and M. T. Siva‐Jothy . 2000 Genital damage, kicking and early death ‐ The battle of the sexes takes a sinister turn in the bean weevil. Nature 407:855–856. 11057654 10.1038/35038154

[ece31775-bib-0020] Eberhard, W. G. 2002 The function of female resistance behavior: intromission by male coercion vs. female cooperation in Sepsid flies (Diptera: Sepsidae). Rev. Biol. Trop. 50:485–505. 12298280

[ece31775-bib-0021] Evans, J. P. 2009 No evidence for sperm priming responses under varying sperm competition risk or intensity in guppies. Naturwissenschaften 96:771–779. 19308348 10.1007/s00114-009-0529-6

[ece31775-bib-0022] Fairbairn, D. J. , and R. F. Preziosi . 1996 Sexual selection and the evolution of sexual size dimorphism in the Water Strider, *Aquarius remigis* . Evolution 50:1549–1559. 10.1111/j.1558-5646.1996.tb03927.x 28565700

[ece31775-bib-0023] Falconer, D. S. , and T. F. C. Mackay . 1996 Introduction to quantitative genetics, 4th ed Prentice Hall, London.

[ece31775-bib-0024] Farr, J. A. 1989 Sexual selection and secondary differentiation in poeciliids: determinants of male mating success and the evolution of female choice Pp. 91–123 *in* Meffe G. K. and Snelson F. F., eds. Ecology and evolution of livebearing fishes (Poeciliidae). Prentice Hall, Upper Saddle River, NJ.

[ece31775-bib-0025] Gavrilets, S. , and G. Arnqvist . 2001 The evolution of female mate choice by sexual conflict. Proc. R. Soc. B 268:531–539. 10.1098/rspb.2000.1382 PMC1088637 11296866

[ece31775-bib-0026] Gerhardt, H. C. , and R. Brooks . 2009 Experimental analysis of multivariate female choice in gray treefrogs (*Hyla versicolor*): evidence for directional and stabilizing selection. Evolution 63:2504–2512. 19500145 10.1111/j.1558-5646.2009.00746.x PMC2763017

[ece31775-bib-0027] Gershman, S. , M. Delcourt , and H. D. Rundle . 2014 Sexual selection on *Drosophila serrata* male pheromones does not vary with male age or mating status. J. Evol. Biol. 27:1279–1286. 24828752 10.1111/jeb.12407

[ece31775-bib-0028] Greene, E. , B. E. Lyon , V. R. Muechter , L. Ratcliffe , S. J. Oliver , and P. T. Boag . 2000 Disruptive sexual selection for plumage coloration in a Passerine bird. Nature 407:1000–1003. 11069178 10.1038/35039500

[ece31775-bib-0029] Hall, M. D. , L. F. Bussiere , J. Hunt , and R. Brooks . 2008 Experimental evidence that sexual conflict influences the opportunity, form and intensity of sexual selection. Evolution 62:2305–2315. 18540949 10.1111/j.1558-5646.2008.00436.x

[ece31775-bib-0030] Han, C. S. , and R. C. Brooks . 2013 Correlational selection does not explain the evolution of a behavioural syndrome. J. Evol. Biol. 26:2260–2270. 23980636 10.1111/jeb.12223

[ece31775-bib-0031] Head, M. L. , A. K. Lindholm , and R. Brooks . 2008 Operational sex ratio and density do not affect directional selection on male sexual ornaments and behavior. Evolution 62:135–144. 18067568 10.1111/j.1558-5646.2007.00277.x

[ece31775-bib-0032] Head, M. L. , F. Jacomb , R. Vega‐Trejo , and M. D. Jennions . 2015 Male mate choice and insemination success under simultaneous versus sequential choice conditions. Anim. Behav. 103:99–105.

[ece31775-bib-0033] Hunt, J. , C. J. Breuker , J. A. Sadowski , and A. J. Moore . 2009 Male‐male competition, female mate choice and their interaction: determining total sexual selection. J. Evol. Biol. 22:13–26. 19120810 10.1111/j.1420-9101.2008.01633.x

[ece31775-bib-0034] Jormalainen, V. 1998 Precopulatory mate guarding in crustaceans: male competitive strategy and intersexual conflict. Q. Rev. Biol. 73:275–304.

[ece31775-bib-0035] Kahn, A. T. , B. Mautz , and M. D. Jennions . 2010 Females prefer to associate with males with longer intromittent organs in mosquitofish. Biol. Lett. 6:55–58. 19755529 10.1098/rsbl.2009.0637 PMC2817265

[ece31775-bib-0036] Kahn, A. T. , J. D. Livingston , and M. D. Jennions . 2012 Do females preferentially associate with males given a better start in life? Biol. Lett. 8:362–364. 22237504 10.1098/rsbl.2011.1106 PMC3367751

[ece31775-bib-0037] Kokko, H. 2005 Treat ‘em mean, keep ‘em (sometimes) keen: evolution of female preferences for dominant and coercive males. Evol. Ecol. 19:123–135.

[ece31775-bib-0038] Lande, R. , and S. J. Arnold . 1983 The measurement of selection on correlated characters. Evolution 37:1210–1226. 10.1111/j.1558-5646.1983.tb00236.x 28556011

[ece31775-bib-0039] Langerhans, R. B. 2011 Genital evolution Pp. 228–240 *in* Evans J. P., Pilastro A. and Schlupp I., eds. Ecology and evolution of poeciliid fishes. Univ. of Chicago Press, Chicago, IL.

[ece31775-bib-0040] Langerhans, R. B. , C. A. Layman , and T. J. DeWitt . 2005 Male genital size reflects a tradeoff between attracting mates and avoiding predators in two live‐bearing fish species. Proc. Natl Acad. Sci. USA 102:7618–7623. 15894618 10.1073/pnas.0500935102 PMC1140428

[ece31775-bib-0041] LeBas, N. R. , L. R. Hockham , and M. G. Ritchie . 2004 Sexual selection in the gift‐giving dance fly, *Rhamphoyia sulcata*, favors small males carrying small gifts. Evolution 58:1763–1772. 15446428 10.1111/j.0014-3820.2004.tb00459.x

[ece31775-bib-0043] Lockhart, A. B. , P. H. Thrall , and J. Antonovics . 1996 Sexually transmitted diseases in animals: ecological and evolutionary implications. Biol. Rev. 71:415–471. 8761160 10.1111/j.1469-185x.1996.tb01281.x

[ece31775-bib-0044] Lynch, M. , and B. Walsh . 1998 Genetics and analysis of quantitative traits. Sinauer Associates, Sunderland, MA.

[ece31775-bib-0045] Matthews, I. M. , J. P. Evans , and A. E. Magurran . 1997 Male display rate reveals ejaculate characteristics in the Trinidadian guppy *Poecilia reticulata* . Proc. R. Soc. B 264:695–700.

[ece31775-bib-0046] McGlothlin, J. W. , P. G. Parker , V. Nolan , and E. D. Ketterson . 2005 Correlational selection leads to genetic integration of body size and an attractive plumage trait in Dark‐Eyed Juncos. Evolution 59:658–671. 15856707

[ece31775-bib-0047] McPeek, M. A. 1992 Mechanisms of sexual selection operating on body size in the mosquitofish (*Gambusia holbrooki*). Behav. Ecol. 3:1–12.

[ece31775-bib-0048] Muller, M. N. , M. E. Thompson , S. M. Kahlenberg , and R. W. Wrangham . 2011 Sexual coercion by male chimpanzees shows that female choice may be more apparent than real. Behav. Ecol. Sociobiol. 65:921–933.

[ece31775-bib-0049] O'Dea, R. E. , M. D. Jennions , and M. L. Head . 2014 Male body size and condition affects sperm number and production rates in mosquitofish, *Gambusia holbrooki* . J. Evol. Biol. 27:2739–2744. 25403851 10.1111/jeb.12534

[ece31775-bib-0050] Ower, G. D. , K. A. Judge , S. Steiger , K. J. Caron , R. A. Smith , J. Hunt , et al. 2013 Multivariate sexual selection on male song structure in wild populations of sagebrush crickets, *Cyphoderris strepitans* (Orthoptera: Haglidae). Ecol. Evol. 3:3590–3603. 24223293 10.1002/ece3.736 PMC3797502

[ece31775-bib-0051] Pilastro, A. , and A. Bisazza . 1999 Insemination efficiency of two alternative male mating tactics in the guppy (*Poecilia reticulata*). Proc. R. Soc. B 266:1887–1891.

[ece31775-bib-0052] Pilastro, A. , E. Giacomello , and A. Bisazza . 1997 Sexual selection for small size in male mosquitofish (*Gambusia holbrooki*). Proc. R. Soc. B 264:1125–1129.

[ece31775-bib-0053] Pilastro, A. , S. Benetton , and A. Bisazza . 2003 Female aggregation and male competition reduce costs of sexual harassment in the mosquitofish *Gambusia holbrooki* . Anim. Behav. 65:1161–1167.

[ece31775-bib-0054] Plath, M. , J. Parzefall , and I. Schlupp . 2003 The role of sexual harassment in cave and surface dwelling populations of the Atlantic molly, *Poecilia mexicana* (Poeciliidae, Teleostei). Behav. Ecol. Sociobiol. 54:303–309.

[ece31775-bib-0055] Plath, M. , A. M. Makowicz , I. Schlupp , and M. Tobler . 2007 Sexual harassment in live‐bearing fishes (Poeciliidae): comparing courting and noncourting species. Behav. Ecol. 18:680–688.

[ece31775-bib-0056] Price, T. , and T. Langen . 1992 Evolution of correlated characters. Trends Ecol. Evol. 7:307–310. 21236041 10.1016/0169-5347(92)90229-5

[ece31775-bib-0202] R Development Core Team . 2012 R: a language and environment for statistical computing. R Foundation for Statistical Computing, Vienna, Austria.

[ece31775-bib-0057] Sih, A. , M. Lauer , and J. J. Krupa . 2002 Path analysis and the relative importance of male‐female conflict, female choice and male‐male competition in water striders. Anim. Behav. 63:1079–1089.

[ece31775-bib-0058] Soltis, J. , F. Mitsunaga , K. Shimizu , Y. Yanagihara , and M. Nozaki . 1997 Sexual selection in Japanese macaques.1. female mate choice or male sexual coercion? Anim. Behav. 54:725–736. 9299056 10.1006/anbe.1997.0567

[ece31775-bib-0059] Stinchcombe, J. R. , A. F. Agrawal , P. A. Hohenlohe , S. J. Arnold , and M. W. Blows . 2008 Estimating nonlinear selection gradients using quadratic regression coefficients: double or nothing? Evolution 62:2435–2440. 18616573 10.1111/j.1558-5646.2008.00449.x

[ece31775-bib-0060] Tadler, A. 1999 Selection of a conspicuous male genitalic trait in the seedbug *Lygaeus simulans* . Proc. R. Soc. B 266:1773–1777.

[ece31775-bib-0061] Thornhill, R. , and J. Alcock . 1983 The evolution of insect mating systems. Harvard Univ. Press, Cambridge, MA.

[ece31775-bib-0062] Vega‐Trejo, R. , R. E. O'Dea , M. D. Jennions , and M. L. Head . 2014 The effects of familiarity and mating experience on mate choice in mosquitofish, *Gambusia holbrooki* . Behav. Ecol. 25:1205–1211.

[ece31775-bib-0063] Westneat, D. F. , P. W. Sherman , and M. L. Morton . 1990 The ecology and evolution of extra‐pari copulations in birds. Curr. Ornith. 7:331–369.

[ece31775-bib-0064] Wigby, S. , and T. Chapman . 2005 Sex peptide causes mating costs in female *Drosophila melanogaster* . Curr. Biol. 15:316–321. 15723791 10.1016/j.cub.2005.01.051

[ece31775-bib-0065] Wiley, R. H. , and J. Poston . 1996 Indirect mate choice, competition for mates, and coevolution of the sexes. Evolution 50:1371–1381. 10.1111/j.1558-5646.1996.tb03911.x 28565703

[ece31775-bib-0066] Wyman, M. J. , and L. Rowe . 2014 Male bias in distributions of additive genetic, residual, and phenotypic variances of shared traits. Am. Nat. 184:326–337. 25141142 10.1086/677310

[ece31775-bib-0067] Yakami, Y. 2003 Experimental analysis of the effect of genital morphology on insemination success in the ground beetle *Carabus insulicola* (Coleoptera Carabidae). Ethol. Ecol. Evol. 15:51–61.

